# Electroactive nano-Biohybrid actuator composed of gold nanoparticle-embedded muscle bundle on molybdenum disulfide nanosheet-modified electrode for motion enhancement of biohybrid robot

**DOI:** 10.1186/s40580-022-00316-8

**Published:** 2022-05-25

**Authors:** Minkyu Shin, Jin-Ha Choi, Joungpyo Lim, Sungwoo Cho, Taehyeong Ha, Jae Hyun Jeong, Jeong-Woo Choi

**Affiliations:** 1 Department of Chemical & Biomolecular Engineering, Sogang University, Seoul, 04170 Republic of Korea; 2 School of Chemical Engineering, Jeonbuk National University, 567 Baekje-daero, Deokjin-gu, Jeonju-si, Jeollabuk- do 54896 Republic of Korea; 3 Department of Chemical Engineering, Soongsil University, 369, Seoul, 06978 Republic of Korea

**Keywords:** Biohybrid robot, Biorobotic hand, Nano-biohybrid actuator, Muscle bundle, MoS_2_ nanosheet, Electroactive bioactuator

## Abstract

There have been several trials to develop the bioactuator using skeletal muscle cells for controllable biobybird robot. However, due to the weak contraction force of muscle cells, the muscle cells could not be used for practical applications such as biorobotic hand for carrying objects, and actuator of biohybrid robot for toxicity test and drug screening. Based on reported hyaluronic acid-modified gold nanoparticles (HA@GNPs)-embedded muscle bundle on PDMS substrate, in this study for augmented actuation, we developed the electroactive nano-biohybrid actuator composed of the HA@GNP-embedded muscle bundle and molybdenum disulfide nanosheet (MoS_2_ NS)-modified electrode to enhance the motion performance. The MoS_2_ NS-modified Au-coated polyimide (PI) electrode to be worked in mild pH condition for viable muscle cell was utilized as supporting- and motion enhancing- substrate since it was electrochemically active, which caused the movement of flexible PI electrode. The motion performance of this electroactive nano-biohybrid actuator by electrical stimulation was increased about 3.18 times compared with that of only HA@GNPs embedded-muscle bundle on bare PI substrate. The proposed electroactive nano-biohybrid actuator can be applied to the biorobotic hand and biohybrid robot.

**Supplementary information:**

The online version contains supplementary material available at 10.1186/s40580-022-00316-8.

## Introduction

Bio-inspired artificial robot activated by electrical or optical stimulation have been developed with great potential [1–3]. Among them, various types of muscle cell-based biohybrid robots have been developed, which are controlled by electrical or optical stimulation, such as stingrays, jellyfish, and self-propelled swimming robots [4–6]. However, the muscle cells themselves do not have enough contraction force to apply in practical applications. To overcome this problem, various nanomaterials such as gold nanorods, graphene oxide, and carbon nanotubes have been introduced into muscle cells for augmentation of contraction force of muscle cells [7–9]. In addition to enhanced motion performance, these nanomaterials have positive effects on muscle maturation and regeneration [10, 11]. For example, gold nanoparticles (GNPs) have attracted much attention due to their high electrical conductivity, biocompatibility, and induction of differentiation in muscle cells [12–14].

In the neuromuscular system, muscle cells are stimulated by neurotransmitters secreted from neurons for contraction and relaxation [15, 16]. This phenomenon can be induced in the same way by applying extermal electrical stimulation; thus, contraction-relaxation can be controlled by external electrical stimulation. Recently, there is a focus on electrochemical actuators, which convert electrical energy to mechanical energy through the electrochemical reaction, due to advantages such as rapid response and large bending displacement [17–19]. Similar to neuromuscular system, electrochemical actuators can be also controlled by external electrical stimulation [20, 21]. Accordingly, when muscle cells are combined with electrochemical actuators, they can be controlled by electrical stimulation to improve motion performance.

Recently, we reported a hyaluronic acid (HA) modified GNPs (HA@GNPs)-embedded muscle bundle on polydimethylsiloxane (PDMS) substrate for enhancement of muscle cell differentiation and actuation of muscle cells [22]. The maturation and differentiation of muscle cells could be improved owing to the positive effects of GNPs and HA. HA could inhibit the formation of premature myotubes and enhance the recruitment of muscle progenitor cells [12, 23–25]. HA@GNPs-embedded muscle bundle on PDMS substrate was applied to fabricate biohybrid robot to evaluate inotropic effects. However, the HA@GNPs-embedded muscle bundle still has the weak contraction force to be used for bioactuator.

The molybdenum disulfide (MoS_2_) nanosheet (NS)-modified Au-coated polyimide (PI) electrode was developed to be used as the electrochemical actuator at a low voltage, owing to its excellent charge effect and large surface area [21, 26, 27]. However, the MoS_2_ NS-based electrochemical actuator was operated at extremely low pH value (about 0.3) and it could not be conjugated with muscle cells. Thus, there has been no report for the application of a MoS_2_ NS-modified Au-coated PI electrode to bioactuator.

**Fig. 1:**
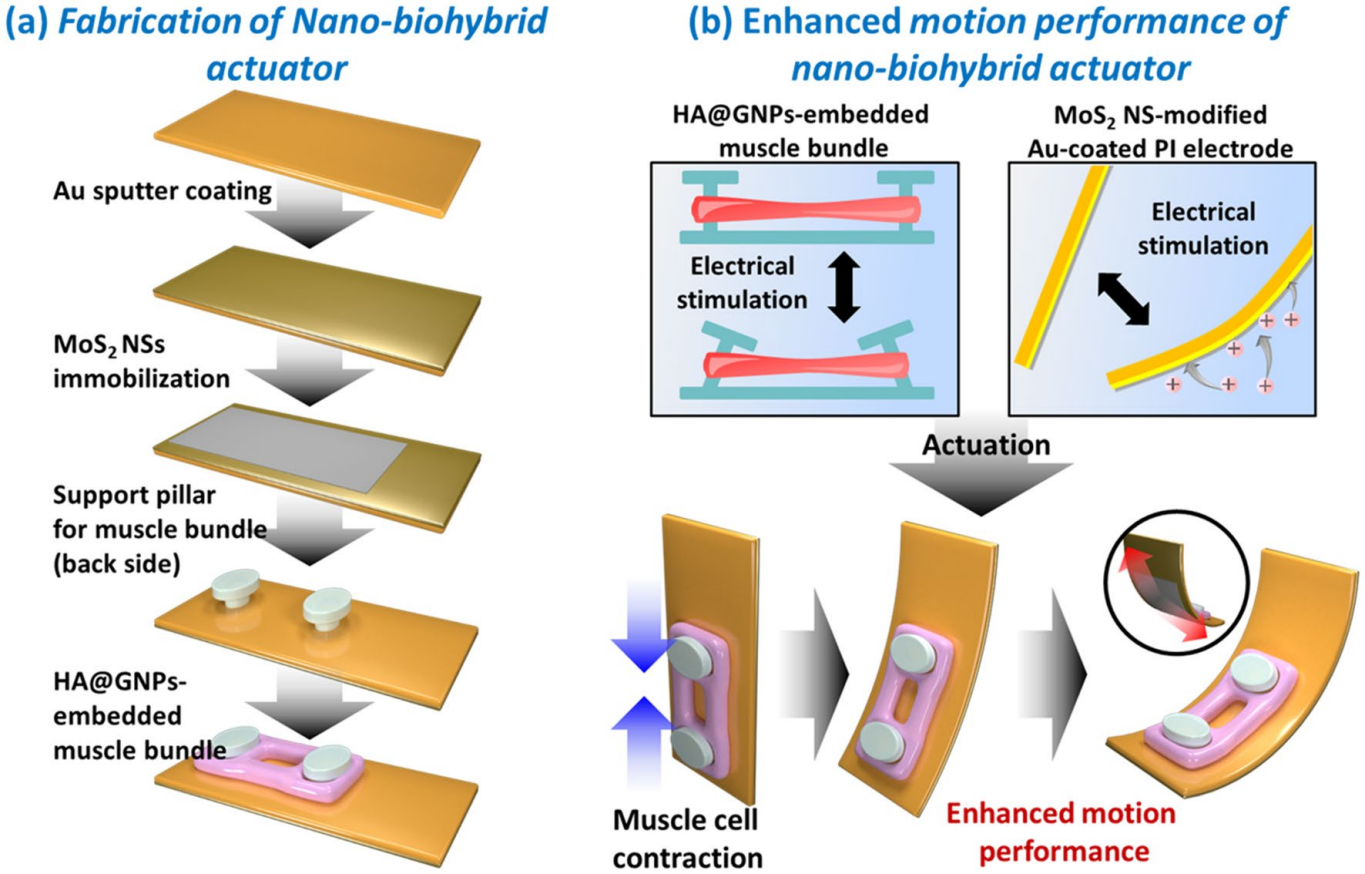
Schematic diagram of electroactive nano-biohybrid actuator fabrication and its actuation mechanism. **a** fabrication of the electroative nano-biohybrid actuator by using HA@GNPs-embedded muscle bundle and MoS_2_ NSs based acutator. **b** preparation of HA@GNPs-embedded muscle bundle and the motion performance of bioacautor

To overcome the weak force of the muscle cells, in this study, an electroactive nano-biohybrid actuator with significantly enhanced motion performance was developed for the first time by integrating a HA@GNPs-embedded muscle bundle with a MoS_2_ NS-modified Au-coated PI electrode (Fig. 1). The actuation of MoS_2_ NS-modified Au-coated PI electrode in PBS electrolyte of neutral pH (about 7.4) was investigated for muscle cell culture. In this study, the MoS_2_ NS was synthesized using chemical exfoliation method to fabricate the MoS_2_ NS-modified Au-coated PI electrode. The MoS_2_ NS-modified Au-coated PI electrode was controlled according to different voltages and times. To develop the electroactive nano-biohybrid actuator, the HA@GNPs-embedded muscle bundle was combined with MoS_2_ NS-modified Au-coated PI electrode using PDMS support pillar (Fig. 1a). As both the HA@GNPs-embedded muscle bundle and MoS_2_ NS-modified Au-coated PI electrode were driven by external low-voltage electrical stimulation, it was easy to synchronize their motion performance and enhance the motion performance. The fabricated electroactive nano-biohybrid actuator can be used as a basic platform for the development of biohybrid robots with augmented force in the future.

## Methods/experimental

### Synthesis of MoS_2_ NS

MoS_2_ NSs were synthesized by the chemically exfoliated method and lithium intercalation. MoS_2_ powder (2 g) was dissolved in 20 mL of n-butyllithium solution in a flask filled with argon gas for 48 h at 60 °C. After the reaction, the excess lithium and residue were centrifuged at 10,000 rpm for 30 min and washed with hexane. MoS_2_ NSs were obtained through deintercalation of the lithium ions present between the MoS_2_ layers by ultrasonication of the obtained precipitate in deionized water (DIW) for 1 h. To remove the non-exfoliated MoS_2_, the resulting MoS_2_ NS solution was centrifuged at 2000 rpm for 30 min, and the supernatant was centrifuged at 10,000 rpm for 30 min. The MoS_2_ NSs precipitated through centrifugation were dissolved in DIW and stored at a concentration of 10 mg/mL. The synthesized MoS_2_ NSs were characterized by high-resolution TEM using a JEOL JEM-3010 microscope operated at 300 kV, energy-dispersive X-ray spectroscopy (EDS), and atomic force microscopy (AFM). In addition, a carbon TEM grid (No. 01811; Ted Pella Inc., Redding, CA, USA) was used for TEM analysis.

### Fabrication of MoS_2_ NS-modified Au-coated PI electrode

To fabricate the MoS_2_ NS-modified Au-coated PI electrode, a polyimide (PI) film (25 μm thickness) was used as the support fixture. The PI film was washed with acetone in a sonication bath for 30 min and washed once more with DIW for 30 min, followed by complete drying with nitrogen gas. The prepared PI film was Au sputter-coated to form a 100 nm-thick Au layer. Then, an 8 μm-thick MoS_2_ layer, which was prepared by vacuum filtration using a nitrocellulose membrane, was attached to the Au layer. The 8 μm-thick MoS_2_ layer was prepared using 40 mL of 2 mg/mL MoS_2_ NS solution. To transfer the fabricated MoS_2_ layer to the Au-coated PI film, PMMA was spin coated on the filtered MoS_2_ layer on the membrane at 2000 rpm for 60 s, followed by baking at 50 °C for 30 min. Then, 3 M KOH solution was prepared by dissolving KOH pellets in DIW. The PMMA-coated MoS_2_ layer was placed in the KOH solution, and it was left in the solution until it was separated from the membrane. The separated PMMA-coated MoS_2_ layer was transferred from the KOH solution to DIW. The PMMA-coated MoS_2_ layer was transferred onto the Au-coated PI film. Finally, PMMA was dissolved in acetone, followed by drying in a 70 °C oven for a week. The structure of the MoS_2_ NS-modified Au-coated PI electrode was confirmed by Field Emission-Scanning Electron Microscopy (FE-SEM; S-4700, Hitachi, JP) at the Future Energy Convergence Core Center (FECC).

### Fabrication of HA@GNPs-embedded muscle bundle for biohybrid actuator

To fabricate the HA@GNPs-embedded muscle bundle for the biohybrid actuator, GNPs were modified with HA (HA@GNPs) for muscle cell differentiation promotion (Additional file 3: Fig. S1a). In brief, HA (100 mg) and cystamine dihydrochloride (60 mg) were mixed with 10 mL of 0.1 M boric acid buffer and 0.4 M NaCl for 2 h. Then, 200 mM NaBH_3_CN was added to the resulting solution and stirred for 5 days at 40 °C. After the reaction, 100 mM dithiothreitol (DTT) was added to the solution to introduce a free thiol group. Thiol-modified HA was synthesized by washing the solution through dialysis (MWCO: 3500 Da) against an excess of 100 mM NaCl solution for 2 days, 25% ethanol for 1 day, and DIW for 1 day, followed by freeze drying for 3 days. For the fabrication of HA@GNPs, 10 mL of GNP solution was mixed with 2 mg of thiol-modified HA for 24 h. Then, the HA@GNPs were collected by centrifugation at 400 rpm for 45 min. The HA@GNPs were examined by transmission electron microscopy (TEM) and UV-vis to confirm successful synthesis (Additional file 3: Fig. S1, b and c). To fabricate the HA@GNPs-embedded muscle bundle, C2C12 cells were mixed with HA@GNPs using an extracellular matrix (ECM) hydrogel. In brief, the C2C12 cells were mixed at a concentration of 5 × 10^6^ cells/mL with the ECM hydrogel containing 30% Matrigel, fibrinogen (4 mg/mL), thrombin (2 U/mL), and HA@GNPs (3 nM). Then, 200 µL of the prepared HA@GNPs-embedded muscle bundle was loaded into the PDMS mold and incubated for 30 min for gelation. C2C12 cells were maintained in growth medium consisting of high-glucose DMEM supplemented with 10% FBS and 1% penicillin/streptomycin at 37 °C with 5% CO2. The loaded HA@GNPs-embedded muscle bundle was differentiated in DMEM containing 2% horse serum, 1 mg/mL aminocaproic acid, 1 ng/mL insulin growth factor-1, and 1% penicillin/streptomycin. The differentiation medium was replaced every 2 days for 2 weeks.

### Electrochemical properties of the MoS_2_ NS-modified Au-coated PI electrode

The electrochemical properties of the prepared MoS_2_ NS-modified Au-coated PI electrode and biohybrid actuator were examined using the electrochemical analyzer CHI-660E (CH Instruments Inc., Bee Cave, TX, USA). A multi-potential step technique was used with a three-electrode system composed of the prepared actuator as a working electrode, a platinum (Pt) wire counter electrode, and a silver/silver chloride (Ag/AgCl) reference electrode. The electrochemical experiments were conducted with a mixture of PBS electrolyte (pH = 7.4) as the electrolyte for the actuator. An electrochemical experiment with the multi-potential step technique was performed by applying various voltages for the same time period and by applying the same voltage for different time periods. The parameters of the experiment with various voltages for the same time period were as follows: step time of 5 s, quiet time of 2 s, sensitivity of 1 × 10^1^ A/V, and step E of ± 0.3 V to ± 0.8 V. The parameters of the experiment with the same voltage for different time periods were as follows: step E of ± 0.5 V, quiet time of 2 s, sensitivity of 1 × 10^1^ A/V, and step time of 1 to 10 s. The data obtained through the multi-potential step technique were fitted through origin.

## Results and discussion

### Characterization of MoS_2_ NSs electrode as an electrochemical actuator

**Fig. 2:**
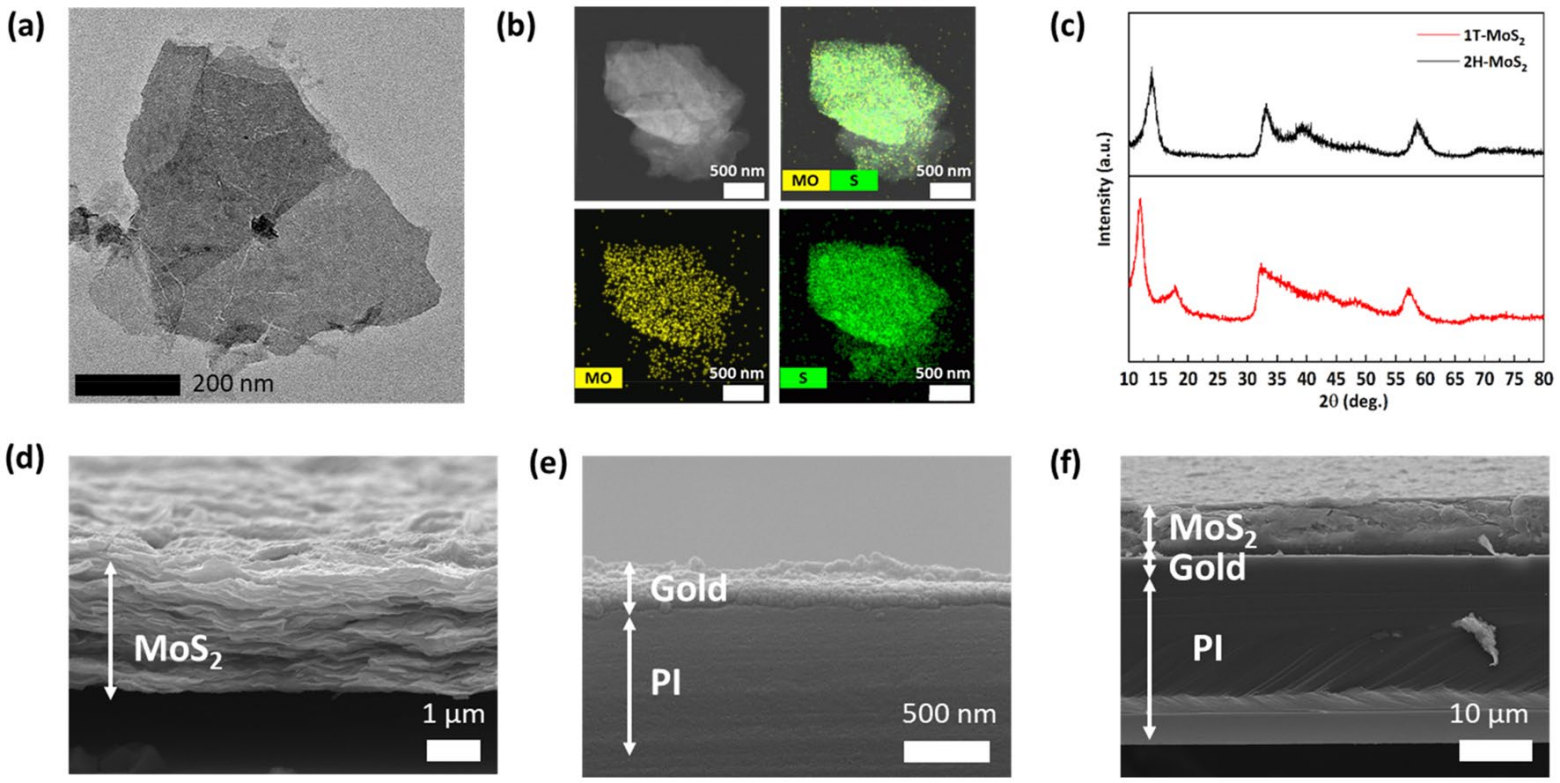
Fabrication of MoS_2_ NSs based actuator. **a** TEM image and **b** EDS mapping image of MoS_2_ NSs. **c** XRD pattern analysis of MoS_2_ NSs. Cross-sectional SEM images of **d** the vacuum filtered MoS_2_ NSs, **e** gold coated PI film and **f** MoS_2_ NS-modified Au-coated PI electrode

MoS_2_ NSs were synthesized to fabricate the electrochemical actuator for augmentation of motion performance. The conformation and composition of the synthesized MoS_2_ NSs were examined by TEM, EDS, and X-ray crystallography (XRD) analysis. As shown in Fig. 2a, the TEM image revealed that the synthesized MoS_2_ NSs had a thin-layer two-dimensional structure. The EDS mapping images are shown in Fig. 2b. The images demonstrated that the ratio of molybdenum (Mo) to sulfur (S) was approximately 1:2. XRD diffractometer analysis of MoS_2_ NSs was conducted at a scanning rate of 5°/min and with a 2θ range from 10° to 80°. The XRD pattern of 2 H-MoS_2_ showed peaks at 14.42°, 33.18°, 39.14°, and 58.56°, corresponding to the (002), (100), (103), and (110) planes of hexagonal MoS_2_ (JCPDS card no. 34-1492). In comparison with 2 H-MoS_2_, the (002) peak of the synthesized MoS_2_ NSs was shifted to 11.85°, indicating that the NSs were synthesized in the octahedral 1T phase with excellent electrical properties (Fig. 2c). In addition, to measure the thickness of MoS_2_ NSs synthesized by the chemically exfoliated method, 1 mg/mL MoS_2_ NS solution was dropped on a mica substrate and dried at room temperature. The prepared MoS_2_ NSs were examined by AFM (Additional file 3: Fig. S2a and b). The AFM results were investigated with an aspect ratio of 1:1, a proportional gain of 0.5, an integral gain of 0.25, a scan rate of 0.999 Hz, and an amplitude set point of 0.2 V. As shown in Additional file 3: Fig. S2b, the thickness of MoS_2_ NSs was around 1 nm, and it was confirmed that MoS_2_ in bulk form was effectively converted to the NS form through the deintercalation of lithium ions. The MoS_2_ NS-modified Au-coated PI electrode was fabricated using MoS_2_ NSs as shown in Additional file 3: Fig. S2c, and the fabrication of the MoS_2_ NS-modified Au-coated PI electrode was confirmed by cross-sectional SEM analysis. A cross-sectional SEM image of the vacuum-filtered MoS_2_ NSs is shown in Fig. 2d. In the fabricated MoS_2_ layer, MoS_2_ NSs (2 nm) were layered by vacuum filtration to a thickness of 3 μm. The uniform 100 nm-thick Au coating on the PI film is shown in Fig. 2e. The final actuator was prepared by transferring an 8 μm-thick MoS_2_ layer onto the Au-coated PI film using PMMA (Fig. 2f and Additional file 3: Fig. S2d).

### Electrochemical properties and motion performance of the MoS_2_ NS-modified Au-coated PI electrode

**Fig. 3:**
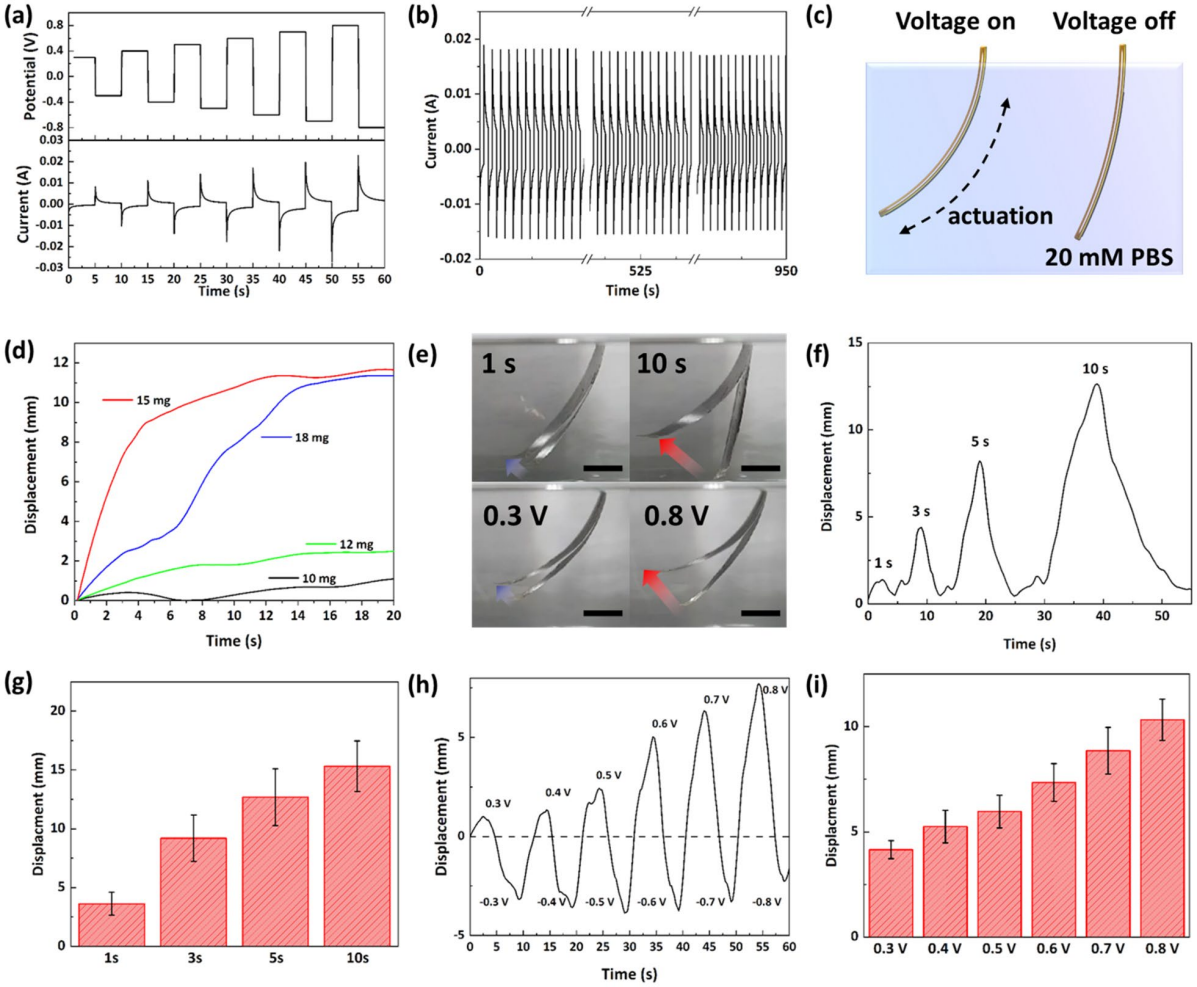
Confirmation of actuator motion performance. **a** variation of current value with applied potential. **b** cycling stability of MoS_2_ NS-modified Au-coated PI electrode. **c** schematic diagram of actuation of MoS_2_ NS-modified Au-coated PI electrode in 20 mM PBS. **d** displacement of MoS_2_ NS-modified Au-coated PI electrode of different weight of MoS_2_ NSs. **e** optical images of actuation in forward direction at 1 s, 10 s, 0.3 and 0.8 V (Scale bar = 5 mm). **f**, **g** displacement of MoS_2_ NS-modified Au-coated PI electrode with diverse time (1, 3, 5, 10 s). **h**, **i** displacement of MoS_2_ NS-modified Au-coated PI electrode with diverse potential (± 0.3, ± 0.4, ± 0.5, ± 0.6, ± 0.7, ± 0.8 V)

The electrochemical actuator is driven by shrinkage and expansion due to ion intercalation between layered structures of MoS_2_ NSs [21, 28, 29]. The electrochemical actuator generally operates based on an acidic solution, but the properties of fabricated MoS_2_ NS-modified Au-coated PI electrode were verified using 20 mM PBS electrolyte to connect to the HA@GNPs-embedded muscle bundle. To verify the biocompatibility of PBS electrolyte, 3-(4,5-dimethylthiazol-2-yl)-2,5-diphenyltetrazolium bromide (MTT) assays were performed (Additional file 3: Fig. S3). The multi-potential step technique was used to verify the electrochemical properties and motion performance of the fabricated MoS_2_ NS-modified Au-coated PI electrode. To measure the current according to the voltage applied to the fabricated MoS_2_ NS-modified Au-coated PI electrode, various voltages (0.3, 0.4, 0.5, 0.6, 0.7, 0.8 V) were applied using the multi-potential step technique. As shown in Fig. 3a, the current was increased linearly with an increasing voltage. The current showed a trend similar to that of the displacement of the actuator. As the applied voltage was increased, the charge of the MoS_2_ NSs was increased. In addition, the stability of the actuator was determined based on the electrochemical properties of the MoS_2_ NS-modified Au-coated PI electrode. The motion stability test was performed to confirm the stable bending motion of the fabricated electrochemical actuator in 20 mM PBS electrolyte. The actuation was stable in a continuous bending motion over 100 cycles as shown in Fig. 3b. The multi-potential step technique was used to evaluate the movement of the MoS_2_ NS-modified Au-coated PI electrode according to the time and voltage. In addition, experiments to verify actuator motion were performed using a 1 cm (width) × 2.5 cm (length) actuator. The bending motion of the actuator was generated by ion intercalation when applying the voltage (Fig. 3c). In addition, actuator displacement according to the amount of MoS_2_ NSs was examined. As shown in Fig. 3d, the displacement was similar with 15–18 mg of MoS_2_ NSs, and the greatest movement was observed with 15 mg of MoS_2_ NSs. To measure the displacement of the fabricated MoS_2_ NS-modified Au-coated PI electrode, the tip of the actuator was fixed to the working electrode, and the other end was immersed in a space filled with the electrolyte (20 mM PBS); subsequently, the voltage was applied. Figure 3e shows the change in the motion according to the time and magnitude of the voltage applied to the actuator. The displacement was measured by applying the same voltage of 0.5 V for 1, 3, 5, and 10 s. Due to the excellent charge effect and electrical properties of MoS_2_, the fabricated actuator exhibited sufficient bending motion at a low voltage and a fast reaction speed. Figure 3f and g, shows the bending motion of the actuator when a voltage of + 0.5 V to -0.5 V was applied to the actuator for 1 and 10 s. As the time of the voltage applied to the actuator was increased, the bending angle of the actuator was also increased linearly (0.896 of the coefficient of determination value (R^2^)). In addition, Fig. 3 h and i, shows the bending motion of the actuator when various voltages were applied to the actuator for the same length of time. The displacement was measured while increasing the applied voltage from 0.3 to 0.8 V at intervals of 0.1 V for 5 s. As the voltage applied to the actuator was increased, the displacement of the actuator was also increased linearly (0.987 of the coefficient of determination value (R^2^)).

### Motion performance of the electroactive nano-biohybrid actuator

**Fig. 4:**
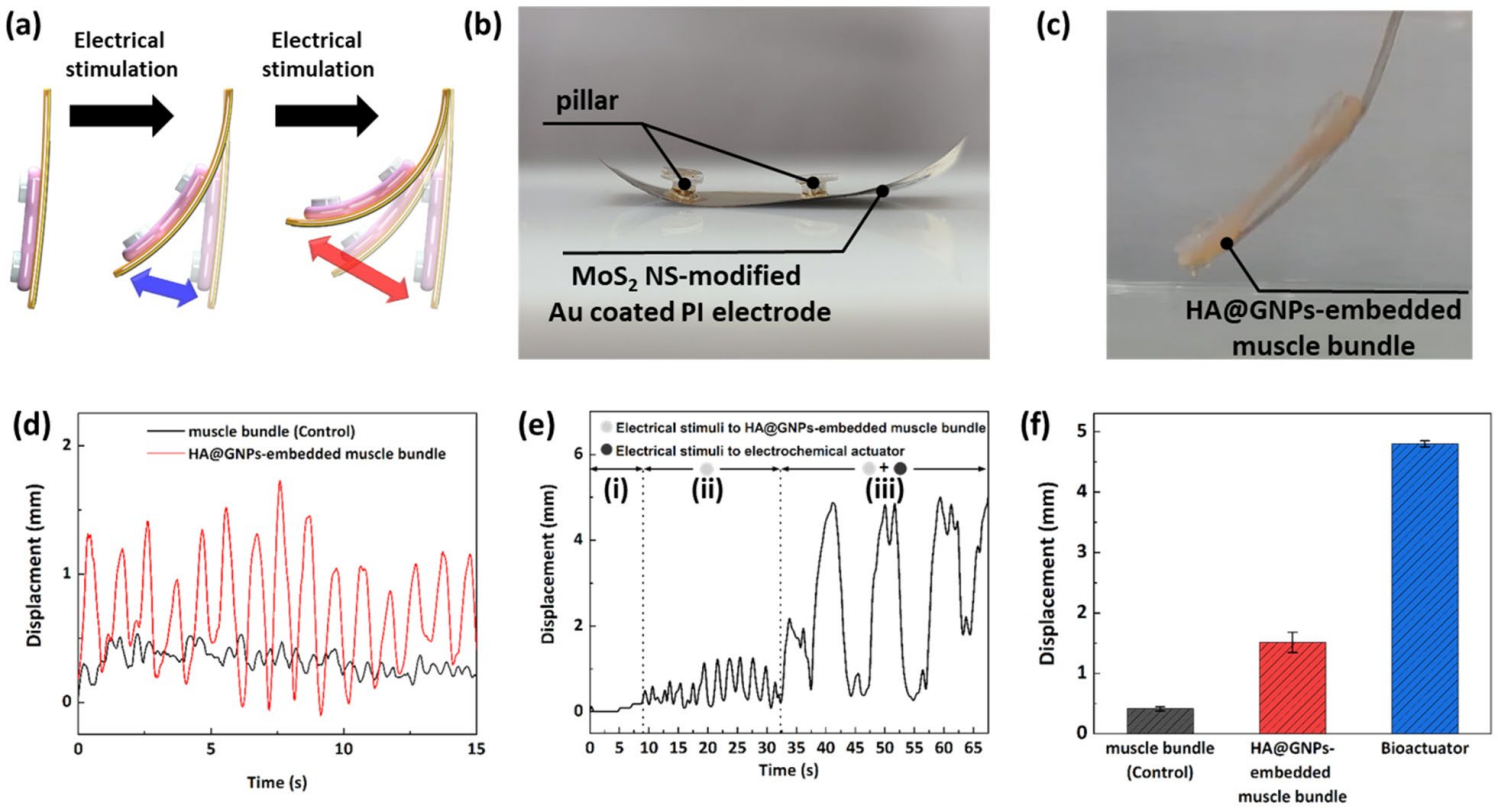
Verification of electroactive nano-biohybrid actuator motion performance. **a** schematic diagram of motion performance of electroactive nano-biohybrid actuator. Optical images of **b** MoS_2_ NS-modified Au-coated PI electrode with pillar and **c** electroactive nano-biohybrid actuator. **d** displacement of HA@GNPs-embedded muscle bundle and without HA@GNPs (control). **e** displacement of electroactive nano-biohybrid actuator (i) without stimulation, (ii) only electrical stimulation, (iii) both electrical and electrochemical stilmulation. **f** displacement of electroactive nano-biohybrid actuator compared with HA@GNPs-embedded muscle bundle

The bending motion of the electroactive nano-biohybrid actuator was generated by combining the contraction of the fabricated HA@GNPs-embedded muscle bundle and the movement of the MoS_2_ NS-modified Au-coated PI electrode (Fig. 4a). To develop the electroactive nano-biohybrid actuator, the fabricated MoS_2_ NS-modified Au-coated PI electrode was combined with the HA@GNPs-embedded muscle bundle using a support pillar. This support pillar was attached to the backside of the MoS_2_ NS-modified Au-coated PI electrode and was used to hang the HA@GNPs-embedded muscle bundle to fabricate the electroactive nano-biohybrid actuator (Fig. 4b and c). The improved differentiation of the HA@GNPs-embedded muscle bundle was confirmed by the immunostaining of muscle differentiation markers. Sarcomeric α-actinin and muscle heavy chain (MHC) were selected as markers to characterize the differentiation of muscle cells (Additional file 3: Fig. S4b). Sarcomeric α-actinin is a cytoskeletal actin-binding protein that maintains the structural integrity of the Z-line of the skeletal muscle [30–32]. MHC is one of the motor proteins in muscle filaments [33, 34]. The expression level of myogenic genes also showed similar results to those for myotube formation. In myogenic differentiation, myogenic factor 5 (*Myf5*) and myogenin (*MyoG*) promote differentiation and muscle-specific gene expression (*MyHC1*). To investigate the expression levels of *Myf5*, *MyoG*, and *MyHC1*, real time-polymerase chain reaction (RT-PCR) was performed using the HA@GNPs-embedded muscle bundle on day 14 (Additional file 3: Fig. S4e-g). The expression levels of *Myf5*, *MyoG*, and *MyHC1* were increased by 3.392, 2.854, and 2.516 times, respectively, which were higher compared with those of the control, HA-embedded, and GNPs-embedded muscle bundle. These results demonstrated that HA and GNPs contained in the HA@GNPs-embedded muscle bundle synergistically activated the differentiation of muscle cells. As the differentiation of muscle cells was improved by HA and GNPs, the movement of HA@GNPs-embedded muscle bundle was enhanced. Also, to confirm the movemet of muscle bundle, HA@GNPs-embedded muslce bundle and muscle bunlde without HA@GNPs were bound to bare PI substrate. The HA@GNPs-embedded muscle bundle on bare PI substrate showed highly enhanced motion performance compared with the performance of the muscle bundle without HA@GNPs (Fig. 4d). Additional file 1: Video S1 shows the contraction displacement of the muscle bundle without HA@GNPs and the HA@GNPs-embedded muscle bundle on bare PI substrate. HA@GNPs activated the differentiation of muscle cells in the muscle bundle and concurrently improved muscle contraction performance. Furthermore, to confirm that the displacement of the electroactive nano-biohybrid actuator was improved by the MoS_2_ NS-modified Au-coated PI electrode, the HA@GNPs-embedded muscle bundle and the MoS_2_ NS-modified Au-coated PI electrode were stimulated. Electrical stimulation was applied to move the HA@GNPs-embedded muscle bundle and the MoS_2_ NS-modified Au-coated PI electrode (Fig. 4e). As shown in Fig. 4e, f, and Additional file 2: Video S2, when electrical stimulation was applied, only the HA@GNPs-embedded muscle bundle was driven with a displacement of approximately 1.5 mm. In addition, when electrical stimulation was simultaneously applied to the MoS_2_ NS-modified Au-coated PI electrode (0.5 V, 5 s) and the HA@GNPs-embedded muscle bundle (20 V, 1 Hz), the HA@GNPs-embedded muscle bundle and the electrochemical actuator moved simultaneously, showing an improved displacement of approximately 4.8 mm.

## Conclusions

In this study, an electroactive nano-biohybrid actuator composed of a HA@GNPs-embedded muscle bundle and a MoS_2_ NS-modified Au-coated PI electrode was fabricated for the enhancement of muscle contraction performance. The myogenic differentiation of cells in the HA@GNPs-embedded muscle bundle was confirmed by measuring the expression levels of *Myf5*, *MyoG*, and *MyHC1*, which were significantly improved by 3.392, 2.854, and 2.516 times, respectively. Due to the introduction of HA@GNPs, the differentiation and contraction performance of the muscle bundle were enhanced by 3.65 times. Moreover, the contraction performance of the muscle bundle was greatly improved with the integration of the MoS_2_ NS-modified Au-coated PI electrode. The synthesized MoS_2_ NSs had a thin layer of around 1 nm, and the ratio of Mo to S was approximately 1:2. In addition, a multi-potential step technique was used to induce the motion of the electroactive nano-biohybrid actuator. The fabricated MoS_2_ NS-modified Au-coated PI electrode showed a linear displacement with respect to the time and voltage, and the motion was stable over 100 cycles. Furthermore, the fabricated electroactive nano-biohybrid actuator was driven by electrical stimulation, and the displacement was 4.8 mm, which was 3.18 times greater compared with that of the HA@GNPs-embedded muscle bundle on PI substrate. Based on the results of actuation enhancement, the proposed electroactive nano-biohybrid actuator can be used as the biorobotic hand and actuation module of biohybrid robot.

## Supplementary Information

**Additional file 1: Video S1**. Contractions of muscle bundle (control) and HA@GNPs-embedded muscle bundle on the bare PI substrate.

**Additional file 2: Video S2**. Motion performance of nano-biohybrid actuator.

**Additional file 3: Figure S1**. Characterization of HA@GNPs-embedded muscle bundle. (a) Fabrication process HA@GNPs-embedded muscle bundle. TEM images of (b) GNP and (c) HA@GNP. (d) optical image of HA@GNPs-embedded muscle bundle. **Figure S2**. Fabrication of MoS_2_ NS-modified Au-coated PI electrode. (a) AFM image and (b) thickness of synthesized MoS_2_ NSs. (c) schematic diagram of synthesis of MoS_2_ NNs and MoS_2_ NS-modified Au-coated PI electrode. (d) optical image of MoS_2_ NS-modified Au-coated PI electrode. **Figure S3**. Analysis to investigate the biocompatibility of the PBS electrolyte for electroactive nano-biohybrid actuator. (a) Quantification of cell viability via MTT assay after 15 min. (b) variation of current value with different electrolyte. **Figure S4**. Confirmation of muscle cell differentiation included in the HA@GNPs-embedded muscle bundle. (a) schematic diagram of composition of HA@GNPs-embedded muscle bundle. (b) immunostainning image of muscle cells cultured on control and HA@GNPs contained hydrogel. Green:α-actinin; blue: hoechst for staining cell nuclei: red:MHC. Scale bar : 100 µm. Morphometric analysis of (c) myotube width and (d) fusion index (*p <= 0.05, **p <= 0.01, and ***p < 0.001). Gene expression levels of (e) myogenic factor 5 (Myf5), (f) myogenin (MyoG), (g) MHC (MyHC1) analyzed by real-time PCR (*p <= 0.05, **p <= 0.01, and ***p < 0.001).

## Data Availability

The datasets used and/or analyzed during the current study are available from the corresponding author on reasonable request.
