# Supplementary material for: Electroactive nano-Biohybrid actuator composed of gold nanoparticle-embedded muscle bundle on molybdenum disulfide nanosheet-modified electrode for motion enhancement of biohybrid robot
Source: Nano Converg. 2022 May 25;9:24. doi: 10.1186/s40580-022-00316-8 (PMC9133293; doi:10.1186/s40580-022-00316-8)
Supplement: Supplementary file 3 — Additional file 3: Figure S1. Characterization of HA@GNPs-embedded muscle bundle. (a) Fabrication process HA@GNPs-embedded muscle bundle. TEM images of (b) GNP and (c) HA@GNP. (d) optical image of HA@GNPs-embedded muscle bundle. Figure S2. Fabrication of MoS2 NS-modified Au-coated PI electrode. (a) AFM image and (b) thickness of synthesized MoS2 NSs. (c) schematic diagram of synthesis of MoS2 NNs and MoS2 NS-modified Au-coated PI electrode. (d) optical image of MoS2 NS-modified Au-coated PI electrode. Figure S3. Analysis to investigate the biocompatibility of the PBS electrolyte for electroactive nano-biohybrid actuator. (a) Quantification of cell viability via MTT assay after 15 min. (b) variation of current value with different electrolyte. Figure S4. Confirmation of muscle cell differentiation included in the HA@GNPs-embedded muscle bundle. (a) schematic diagram of composition of HA@GNPs-embedded muscle bundle. (b) immunostainning image of muscle cells cultured on control and HA@GNPs contained hydrogel. Green:α-actinin; blue: hoechst for staining cell nuclei: red:MHC. Scale bar : 100 µm. Morphometric analysis of (c) myotube width and (d) fusion index (*p <= 0.05, **p <= 0.01, and ***p < 0.001). Gene expression levels of (e) myogenic factor 5 (Myf5), (f) myogenin (MyoG), (g) MHC (MyHC1) analyzed by real-time PCR (*p <= 0.05, **p <= 0.01, and ***p < 0.001). [file 40580_2022_316_MOESM3_ESM.docx]

Supporting Information

**Electroactive Nano-Biohybrid Actuator composed of Gold Nanoparticle-embedded Muscle Bundle on Molybdenum Disulfide Nanosheet-modified Electrode for Motion Enhancement of Biohybrid Robot**

Minkyu Shin^1,†^, Jin-Ha Choi^2, †^, Joungpyo Lim^1^, Sungwoo, Cho^3^, Taehyeong Ha^1^, Jae Hyun Jeong^3,*^ ,and Jeong-Woo Choi^1,*^

^1^ Department of Chemical & Biomolecular Engineering, Sogang University, Seoul 04170, Republic of Korea

^2^ School of Chemical Engineering, Jeonbuk National University, 567 Baekje-daero, Deokjin-gu, Jeonju-si, Jeollabuk-do 54896, Republic of Korea

^3^ Department of Chemical Engineering, Soongsil University, 369, Seoul 06978, Republic of Korea

*Correspondence: Jeong-Woo Choi, Jae Hyun Jeong

† Minkyu Shin and Jin-Ha Choi have equally contributed to this work

Tel.: +82-2-705-8480

E-mail address: jwchoi@sogang.ac.kr, nfejjh@ssu.ac.kr

**TABLE OF CONTENTS FOR SUPPORTING INFORMATION**

**A. CAPTIONS FOR VIDEO CLIPS**

**Video S1** Contractions of muscle bundle (control) and HA@GNPs-embedded muscle bundle on the bare PI substrate.

**Video S2** Motion performance of nano-biohybrid actuator.

**B. SUPPLEMENTARY FIGURES**

**Figure S1** Characterization of HA@GNPs-embedded muscle bundle. (a) Fabrication process HA@GNPs-embedded muscle bundle. TEM images of (b) GNP and (c) HA@GNP. (d) optical image of HA@GNPs-embedded muscle bundle.

**Figure S2** Fabrication of MoS2 NS-modified Au-coated PI electrode. (a) AFM image and (b) thickness of synthesized MoS2 NSs. (c) schematic diagram of synthesis of MoS2 NNs and MoS2 NS-modified Au-coated PI electrode. (d) optical image of MoS2 NS-modified Au-coated PI electrode

**Figure S3** Analysis to investigate the biocompatibility of the PBS electrolyte for electroactive nano-biohybrid actuator. (a) Quantification of cell viability via MTT assay after 15 min. (b) variation of current value with different electrolyte.

**Figure S4** Confirmation of muscle cell differentiation included in the HA@GNPs-embedded muscle bundle. (a) schematic diagram of composition of HA@GNPs-embedded muscle bundle. (b) immunostainning image of muscle cells cultured on control and HA@GNPs contained hydrogel. Green:α-actinin; blue: hoechst for staining cell nuclei: red:MHC. Scale bar : 100 µm. Morphometric analysis of (c) myotube width and (d) fusion index (*p <= 0.05, **p <= 0.01, and ***p < 0.001). Gene expression levels of (e) myogenic factor 5 (Myf5), (f) myogenin (MyoG), (g) MHC (MyHC1) analyzed by real-time PCR (*p <= 0.05, **p <= 0.01, and ***p < 0.001).

**1. Experimental details**

**1.1 Materials**

Bulk MoS_2_ from Sigma-Aldrich (St. Louis, MO, USA) and n-butyllithium from Acros Organics (Thermo Fisher Scientific, Waltham, MA, USA) were used to synthesize the MoS_2_ NS. Poly(methyl methacrylate) (PMMA) was purchased from Microchem, and n-hexane was purchased from Daejung Chemicals & Metals (Korea). Phosphate-buffered saline (PBS) (pH 7.4, 10 mM) was used as the electrolyte in this study. The Sylgard 184 silicone elastomer curing agent and Sylgard 184 silicone elastomer base were acquired from Dow Corning (Midland, MI, USA) for polydimethylsiloxane (PDMS) preparation. All aqueous solutions were prepared using deionized water (DIW) from a Millipore Milli-Q water purifier operating at a resistance of 18 MΩ/cm. Dulbecco’s modified Eagle’s medium (DMEM) from Welgene (Daegu, Korea), fetal bovine serum (FBS) from Young In Frontier (Seoul, Korea), and penicillin from Gibco (Thermo Fisher Scientific, Waltham, MA, USA) were used for media preparation. Anti-sarcomeric α-actinin antibody (Abcam, UK) and MHC antibody (R&D Systems, USA) were used as primary antibodies. Hoechst 33342 from Thermo Scientific (Waltham, MA, USA) and 4% paraformaldehyde from Biosesang (Seongnam, Korea) were used for cell staining. The mouse myoblast cell line C2C12 was purchased from the American Type Culture Collection.

**1.2 Immunostained image of the HA@GNPs-embedded muscle bundle**

The HA@GNPs-embedded muscle bundle was fixed with 4% formaldehyde solution for 10 min at room temperature. After three washes with DPBS, the cells were incubated with primary antibodies overnight at 4 °C. The following primary antibodies were used: sarcomeric α-actinin (1:100) and MHC (1:100). After primary antibody culture, the HA@GNPs-embedded muscle bundle was washed with DPBS and incubated with secondary antibodies (FITC goat anti-rabbit IgG (1: 100) and Texas Red goat anti-mouse IgG (1:100)) overnight at 4 °C. The nuclei were stained with Hoechst (3 μg/mL) for 3 min. The stained HA@GNPs-embedded muscle bundle was washed with DPBS for 10 min. Fluorescent images of the skeletal myocytes of the HA@GNPs-embedded muscle bundle were obtained using a confocal laser scanning microscope. Confocal images were analyzed by Zen black.

**2. Results and Discussion**

**2.1. Differentiation of cells of the HA@GNPs-embedded muscle bundle**

As shown in Fig. S3b, both sarcomeric α-actinin (green) and MHC (red) were highly expressed in the HA@GNPs-embedded muscle bundle with HA@GNPs. In comparison with the control muscle bundle (without HA@GNPs), the skeletal muscle cells of the HA@GNPs-embedded muscle bundle showed significantly matured myotube formation. In addition, the myotube width and fusion index (ratio of the number of nuclei incorporated into myotubes to total number of nuclei) were determined to investigate myotube formation in the HA@GNPs-embedded muscle bundle (Fig. S3c and d). The results showed that the myotube width and fusion index of the HA@GNPs-embedded muscle bundle with HA@GNPs were significantly higher (myotube width: 43.60 μm, fusion index: 64.18%) than those of the control (without HA@GNPs).


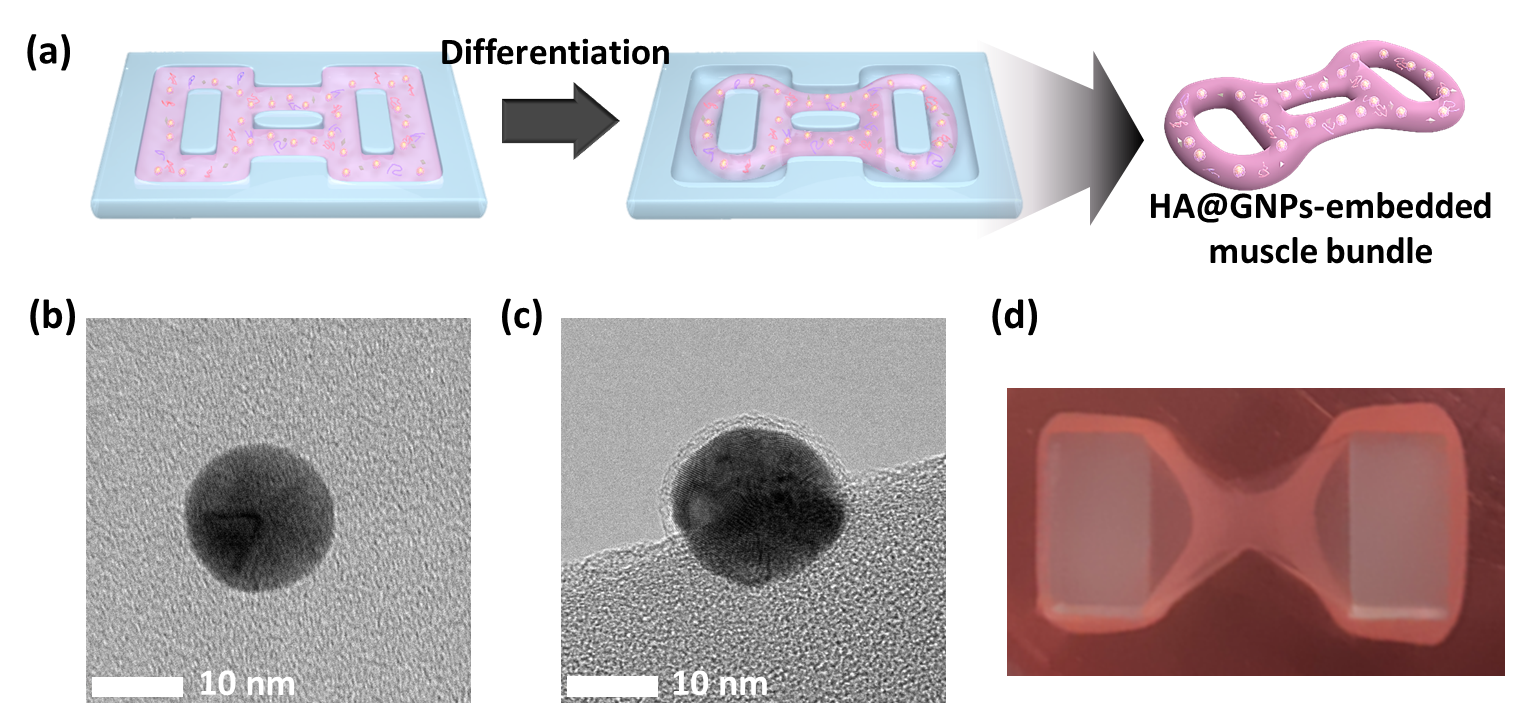


**Figure S1.** Characterization of HA@GNPs-embedded muscle bundle. (a) Fabrication process HA@GNPs-embedded muscle bundle. TEM images of (b) GNP and (c) HA@GNP. (d) optical image of HA@GNPs-embedded muscle bundle.


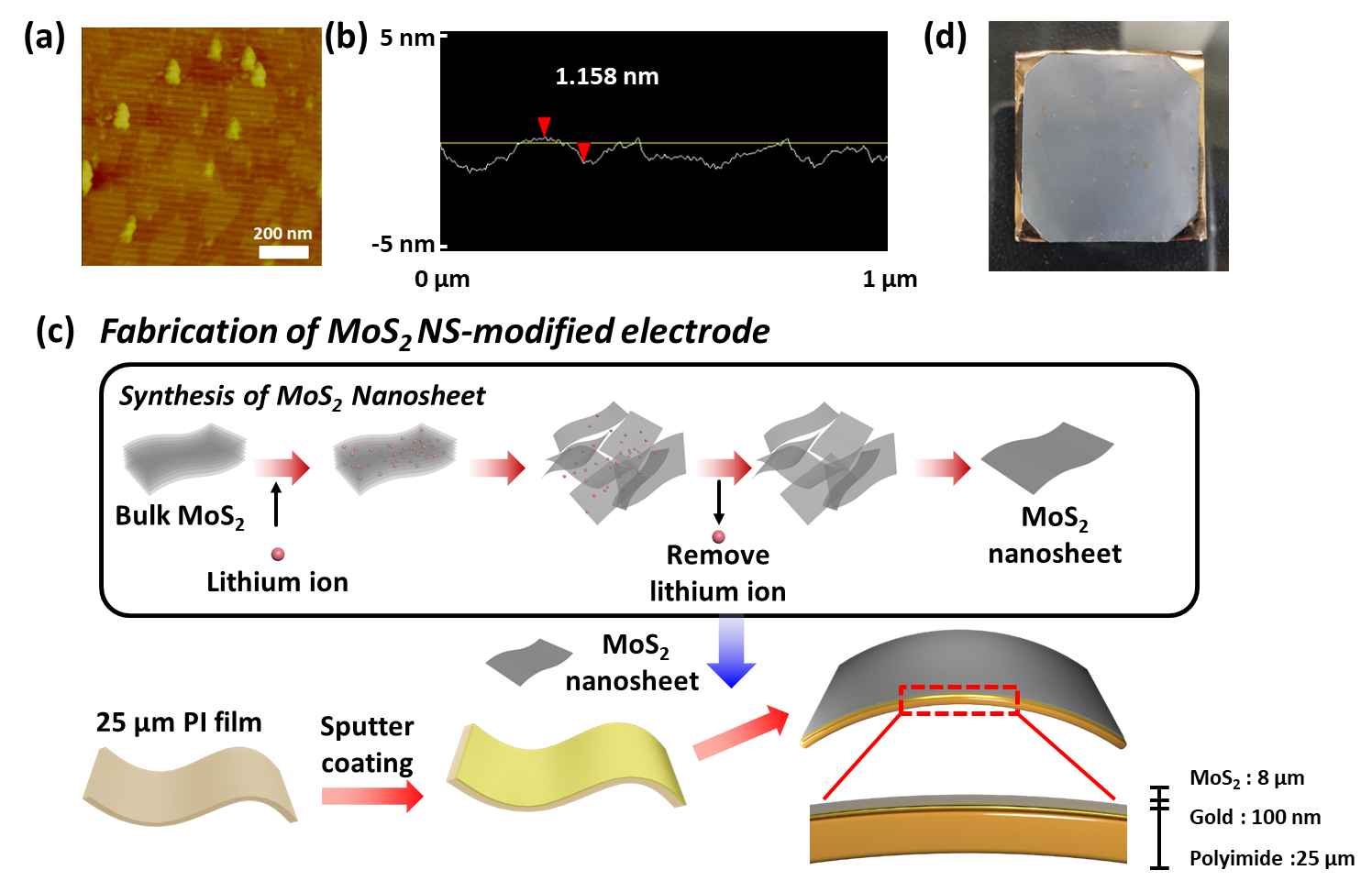


**Figure S2.** Fabrication of MoS_2_ NS-modified Au-coated PI electrode. (a) AFM image and (b) thickness of synthesized MoS_2_ NSs. (c) schematic diagram of synthesis of MoS_2_ NNs and MoS_2_ NS-modified Au-coated PI electrode. (d) optical image of MoS_2_ NS-modified Au-coated PI electrode

**
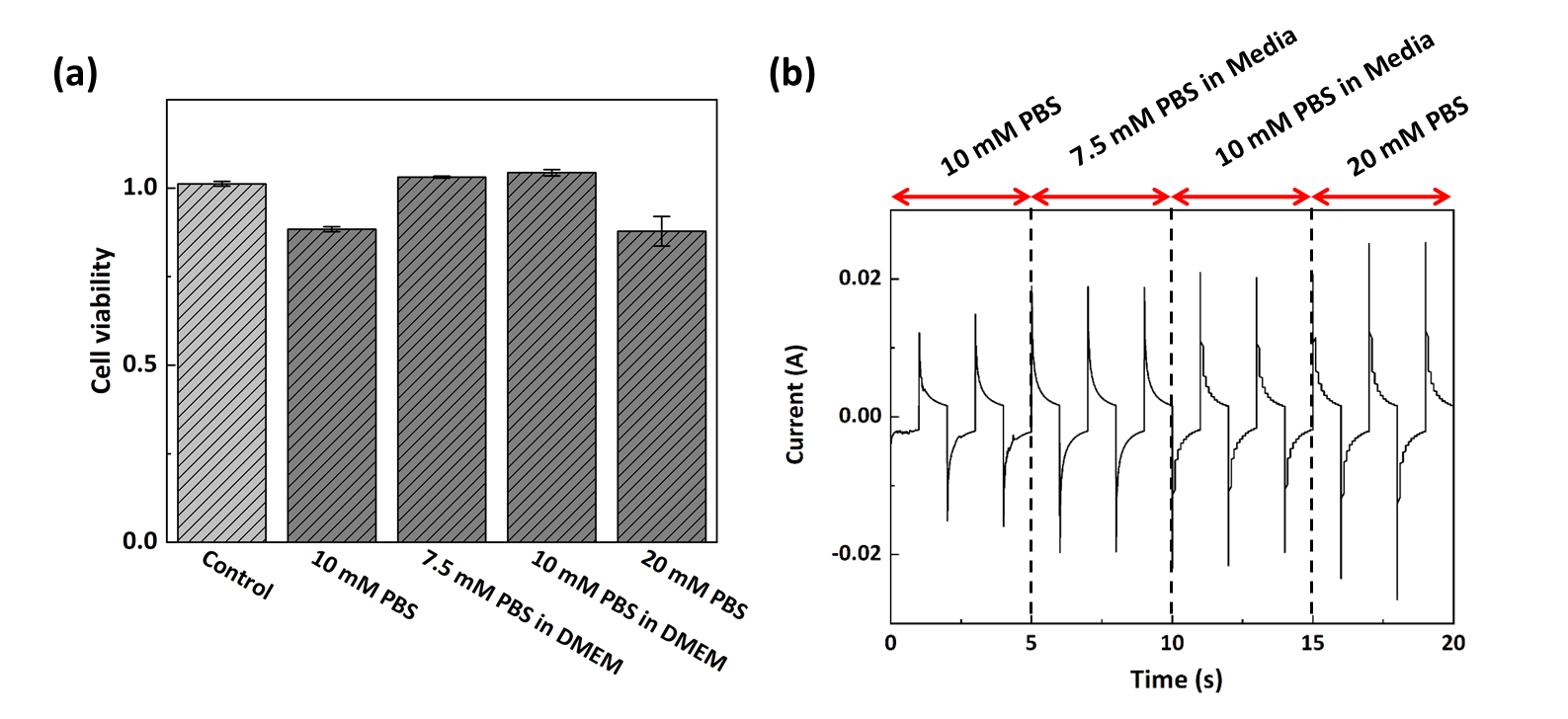
**

**Figure S3.** Analysis to investigate the biocompatibility of the PBS electrolyte for electroactive nano-biohybrid actuator. (a) Quantification of cell viability via MTT assay after 15 min. (b) variation of current value with different electrolyte.


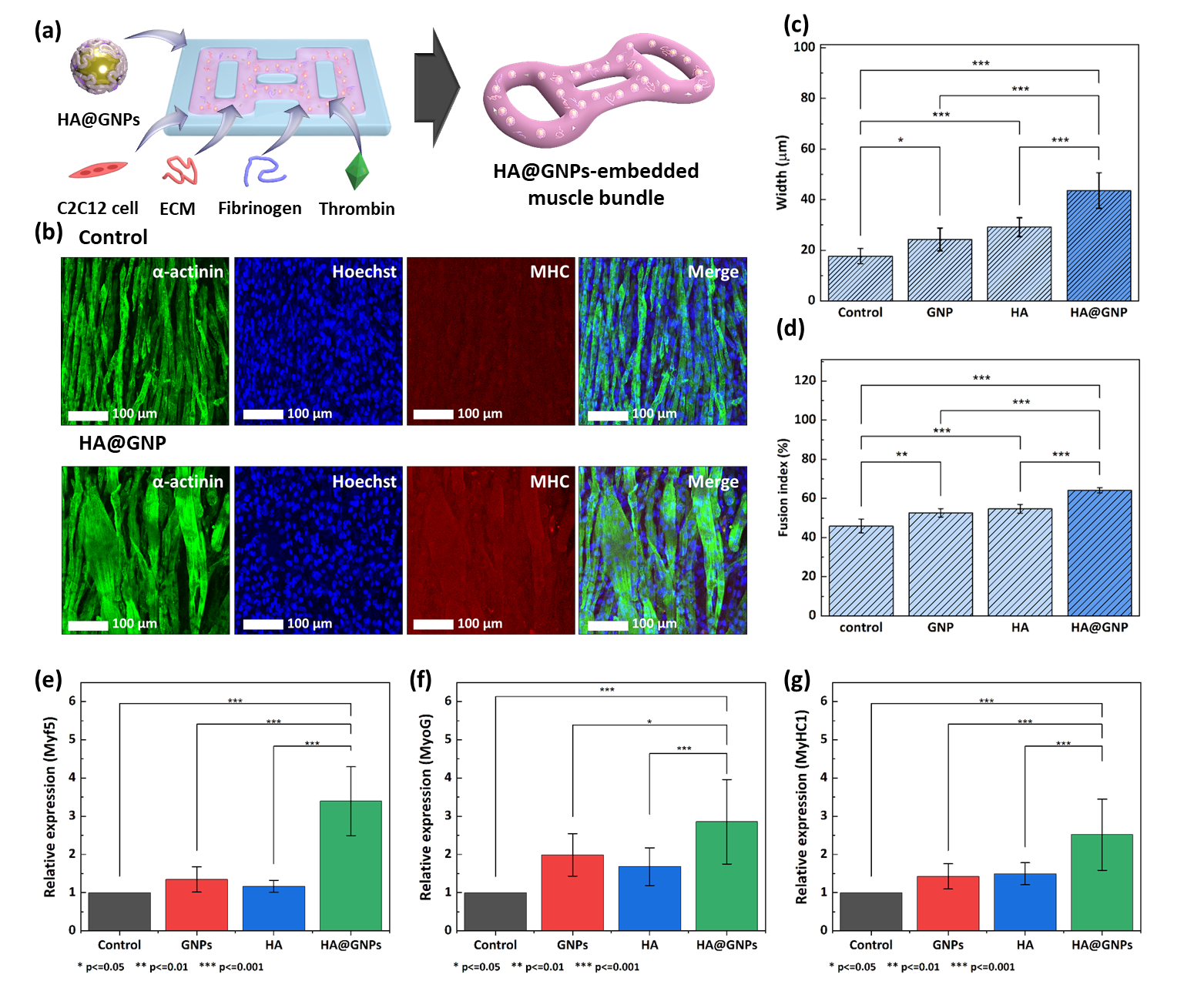


**Figure S4.** Confirmation of muscle cell differentiation included in the HA@GNPs-embedded muscle bundle. (a) schematic diagram of composition of HA@GNPs-embedded muscle bundle. (b) immunostainning image of muscle cells cultured on control and HA@GNPs contained hydrogel. Green:α-actinin; blue: hoechst for staining cell nuclei: red:MHC. Scale bar : 100 µm. Morphometric analysis of (c) myotube width and (d) fusion index (*p <= 0.05, **p <= 0.01, and ***p < 0.001). Gene expression levels of (e) myogenic factor 5 (*Myf5*), (f) myogenin (*MyoG*), (g) MHC (*MyHC1*) analyzed by real-time PCR (*p <= 0.05, **p <= 0.01, and ***p < 0.001).
